# Determinants of health-related quality of life among warfarin patients in Pakistan

**DOI:** 10.1371/journal.pone.0234734

**Published:** 2020-06-17

**Authors:** Muhammad Shahid Iqbal, Fares M. S. Muthanna, Yaman Walid Kassab, Mohamed Azmi Hassali, Fahad I. Al-Saikhan, Muhammad Zahid Iqbal, Abdul Haseeb, Muhammad Ahmed, Salah-Ud-Din Khan, Atta Abbas Naqvi, Md. Ashraful Islam, Majid Ali

**Affiliations:** 1 Department of Clinical Pharmacy, College of Pharmacy, Prince Sattam bin Abdulaziz University, Al-kharj, Saudi Arabia; 2 Faculty of Pharmacy, University Teknologi MARA (UiTM), Selangor Darul Ehsan, Malaysia; 3 Department of Hospital and Clinical Pharmacy, Faculty of Pharmacy, Cyberjaya University College of Medical Sciences, Selangor, Malaysia; 4 Discipline of Social and Administrative Pharmacy, School of Pharmaceutical Sciences, Universiti Sains Malaysia, Penang, Malaysia; 5 Department of Clinical Pharmacy and Pharmacy Practice, Faculty of Pharmacy, AIMST University, Kedah Darul Aman, Malaysia; 6 Department of Clinical Pharmacy, College of Pharmacy, Umm Al-Qura University, Al-Abdia Campus, Makkah, Saudi Arabia; 7 Department of Pharmacology and Toxicology, College of Pharmacy, Umm Al-Qura University, Al-Abdia Campus, Makkah, Saudi Arabia; 8 Department of Biochemistry, College of Medicine, Al-Imam Mohammad Ibn Saud University, Riyadh, Saudi Arabia; 9 Department of Pharmacy Practice, College of Clinical Pharmacy, Imam Abdul Rehman Bin Faisal University, Dammam, Saudi Arabia; St. Paul’s Hospital Millenium Medical College, ETHIOPIA

## Abstract

**Introduction:**

The effect of anticoagulation control on overall Health-Related Quality of Life (HRQoL) in patients taking warfarin in Pakistan has not been explored yet. Therefore, this study aimed to evaluate HRQoL among warfarin patients in Pakistan.

**Methods:**

This cross-sectional study was conducted among patients on warfarin in Pakistan. By purposive sampling, data were collected using demographic data collection form and the World Health Organization Quality of Life: Brief Version (WHOQOL-BREF). The WHOQOL-BREF is comprised of four domains; physical, psychological, social relationships, and environment. Descriptive and inferential statistical analysis was done using SPSS version 22.

**Results:**

Out of 295 warfarin patients, more females than males (<0.001) were observed (n = 184, 62.4%, and n = 111, 37.6% respectively). One hundred and eighteen (40.0%) patients were less than 30-years of age, whereas one hundred and seventy-seven (60.0%) patients were above 30-years of age. Mean scores for the physical (62.44±15.36), psychological (67.84±15.54), social (64.27±26.28) and environment domains (63.45±17.66) were observed.

**Conclusion:**

Patients had overall lower to moderate but satisfactory HRQoL scores in all four domains. Age, gender, employment status, education level, the indication of use and duration of warfarin therapy was associated with one or more domains of HRQoL among warfarin patients. The findings of this study would serve as a primary database for future studies. This study highlights how non-clinical factors could impact HRQoL in studied patients.

## Introduction

"WHO defines Quality of Life as an individual’s perception of their position in life in the context of the culture and value systems in which they live and in relation to their goals, expectations, standards and concerns‴ [1]. Health-Related Quality of Life (HRQoL) is an important measure that enables healthcare professionals (HCPs) to understand patients’ perceptions of illnesses [1–3]. HRQoL is an individual’s perceived Quality of Life (QoL), demonstrating satisfaction in areas of life that are affected by patients’ general health states [4,5]. Furthermore, HRQoL measurement helps in overseeing interventions and making numerous healthcare strategies to amplify the overall health status of a populace [1,2,6].

Warfarin therapy is often used in patients with various cardiovascular diseases (CVDs) like peripheral vascular disease, stroke, atrial fibrillation, and coronary heart disease [7–9]. Studies reported that these CVDs were among the leading causes of premature deaths worldwide. [10–14]. These CVDs are anticipated to affect all aspects of patients’ HRQoL [1,2,15]. Pakistan is also facing serious healthcare issues in CVDs. Various studies show that 1 in 5 of middle-aged adults in Pakistan may develop coronary artery disease, whereas overall CVDs account for approx. 27% of the total deaths [16–18].

In recent decades, the use of oral anticoagulant (OAC) therapy has increased due to its greater efficacy and lesser side effects [4]. Warfarin and heparin have a narrow therapeutic index but high inter and intrapatient variability. Like other anticoagulants, warfarin requires careful and regular laboratory monitoring to help control bleeding complications and achieve superior therapeutic outcomes [19]. In some life-threatening situations, adverse drug reactions caused by warfarin may require immediate hospitalization that if not handled appropriately may lead to morbidity and mortality [20,21]. These prolonged hospital stays expensive therapies and subsequent fear of death negatively affect patients’ daily life activities causing decreased health status [22,23]. Furthermore, warfarin therapy often causes loss of self-esteem, depression, anxiety, failure to perform at the workplace, and emotional problems result in deprived and poor HRQoL [1,2,24].

Similarly, sociodemographic and socioeconomic changes, varied patient care plannings and treatment outcomes also affect HRQoL of patients on warfarin [25–27]. To date, the effect of anticoagulation control on overall HRQoL in patients taking warfarin in Pakistan has not been explored. This study was specially designed to cater to this scarcity and the need for published literature about overall HRQoL of warfarin patients in Pakistan. This study also determined the overall HRQoL of warfarin patients and its relationship with some sociodemographic variables like gender, age, marital and working status, educational level, indication and duration of warfarin use. The study is novel in the aspect that this study first-time documented real-time data of HRQoL among patients on warfarin in Pakistan. Furthermore, as this study is first of its kind and disseminating a primary source of information of HRQoL among warfarin patients in Pakistan so it will serve as the basis for further investigations in the relevant field.

## Material and methods

### Study design and setting

A descriptive and cross-sectional study was conducted among warfarin patients in Pakistan. Though to date, there is no ethical approval requirement for non-clinical and observational studies in Pakistan [28] yet this study was performed following the international clinical guidelines and the principles of the Helsinki Declaration, version 2013 [29]. This study was approved by the Institutional Review Board (IRB) of Clifton Hospital, Karachi Pakistan. All aspects of the study protocol including information on an individual’s background were strictly confidential and used for the research only. Patients were also assured of the confidentiality of their personal information and properly guided about their right to withdraw from the study at any time. All of the participants were ≥18 years and on warfarin from at least 2 months due to any clinical indication and attended the clinic for follow-ups. The period of 2 months is the average time needed to adjust the therapeutic dose of the warfarin [30]. A written consent according to the declaration of Helsinki 1964 and its amendments on comparable ethical standards was taken from all the participants.

Patients who gave written consent and familiar with Urdu (National language of Pakistan) were included in the study. For exclusion criteria, participants who did not give written consent, those from other countries who could not read and understand Urdu, and aged below 18 years were excluded from the study. Pregnant women or those planning to become pregnant were also excluded from the study because, in the first trimester of pregnancy, warfarin therapy can cause fetal anomalies, teratogenicity and fetal miscarriage. In the second trimester, it may lead to premature delivery and low birth weight fetus. In the third trimester, warfarin easily crosses the placenta which may result in fatal maternal bleeding and intracranial hemorrhages [31–33]. The patients having bleeding risks and have uncontrolled INR values i.e. not in the recommended therapeutic range were also excluded.

### Sampling strategy

Purposive sampling was conducted in the hospital and patients undergoing warfarin therapy were identified from the pharmacy’s record. After identification, patients were approached and briefed about the study. Written informed consent was provided to them to indicate their willingness to participate in the study. Those who participated in the study were provided with the questionnaires and their data was noted.

### Sample size calculation

The post-hoc power calculation was conducted using the following formula to determine an adequate sample size.

$$n=\frac{{\left(Z_{1-\beta }\right)}^{2}\left[p(1-p)\right]}{d^{2}}$$

Where, n = required sample size; Z_1-β_ = Z value at power 1-β (at power 95% this value is 1.64); p = referred prevalence, d = margin of error (ideal value is 0.05). Considering 95% power of the test, 5% marginal error and 74% prevalence of warfarin therapy patients within this patient population, in Pakistan [34], the sample size according to the formula was found to be 206.99. The sample size was adjusted for unintentional error/missing rate [35] using the following formula.

$${\text{n}}_{1}=\text{n}/\left(1-\text{d}\right)$$

Where, n = 206.99 and d = 20%. The formula provided a required sample of 258.73 patients. This was the minimum number we obtained from the calculation and we managed to collect 295 complete responses (samples) in our current study.

HRQoL was assessed using the WHOQOL-BREF research tool. This 26 item HRQoL self-administered tool is cross-culturally sensitive which has shown good to excellent psychometric properties [3]. In the WHOQOL-BREF tool’s 26 questions, 2 questions assess the perception of QoL and overall health satisfaction status whereas the other 24 questions comprise the physical, psychological, social and environmental domains. The WHOQOL-BREF tool’s 26 questions explain how respondents attribute to each aspect of their life and how problematic or satisfactory they perceive them to be for their total HRQoL [3]. Demographic characteristics measured were gender, age, marital status, educational level, employment status, comorbidities other than CVDs (diabetes, respiratory diseases, musculoskeletal disorders, pulmonary disorders, and gastrointestinal diseases) indication, and duration of warfarin use. The score of each question for each domain was used to obtain as summarized domain score and finally, all the scores were transformed linearly according to the provided WHOQOL-BREF questionnaire guidelines (0–100 scale) [3]. Higher scores indicate higher levels of HRQoL and vice versa. The Urdu version of the WHOQOL-BREF questionnaire was kindly provided by the WHO officials. A panel of experts reconfirmed the content and face validity of the research instrument in the present study. This study was novel among its types as there was no study evident so far which measured HRQoL among warfarin patients using WHOQOL-BREF in Pakistan.

### Statistical analysis

Descriptive statistics were used to evaluate the demographic and disease characteristics of the patients. Percentages and frequencies were used for the categorical variables, while means and standard deviations were calculated for the continuous variables. The reliability of the WHOQOL-BREF tool was determined using Cronbach’s alpha. The Cronbach’s alpha determines how precisely a set of items, variables or attributes measures a single, one-dimensional latent aspect of participants involved in a study. It is usually used to determine internal consistency (all the items measure the same concept or construct) or inter-item correlations, reliability and statistical power of the items of a research tool for accurate measuring potential research outcomes [36]. To confirm the normality distribution of the data Shapiro–Wilk test and Q-Q plots were used. Independent samples t-test and Spearman’s correlation coefficient was used to evaluate correlations (agreements) between demographics and domains, and to know the differences in overall HRQoL and its domains. By using the linear regression model, predictors were identified and confounders were addressed in the regression model. Data from the questionnaire were analyzed using Statistical Package for the Social Sciences (SPSS) version 22.0.

## Results

In total, 322 respondents filled out the questionnaire in this study. Twenty-seven questionnaires were excluded from the study as they had more than 20% missing data. The remaining 295 respondents were considered for data analysis. The Cronbach’s alpha (reliability) of the WHOQOL-BREF was 0.898. The demographic characteristics of the study participants are presented in Table 1. There was a total of 295 participants with more females than males (n = 184, 62.4%, and n = 111, 37.6%, respectively). One hundred and eighteen (40.0%) were less than 30-years, whereas one hundred and seventy-seven (60.0%) were above 30-years. Ten (3.4%) had a primary level of education and 285 (96.6%) had a higher level of education. One hundred and three (34.9%) had comorbidities other than CVDs and one hundred and ninety-two had no comorbidity.

**Table 1:** Demographic characteristics of the study participants (n = 295).

Description	Frequency	%
**Gender**		
Male	111	37.6
Female	184	62.4
**Age**		
< 30 Years	118	40.0
≥ 30 Years	177	60.0
**Marital Status**		
Single/Separated	115	39.0
Married	180	61.0
**Education Level**		
Primary and secondary	10	3.4
Higher secondary and above	285	96.6
**Work Status**		
Job/Business	181	61.4
Not working	114	38.6
**Comorbidities other than CVDs**		
Yes	103	34.9
No	192	65.1
**Warfarin Indication**		
AF/Valve replacements/Stroke	133	45.1
DVTs/PEs	162	54.9
**Warfarin Duration**		
< 1 Year	126	42.7
≥ 1 Year	169	57.3

Table 2 depicts the individual responses against each item of the questionnaire. Cronbach’s alpha for the whole WHOQOL-BREF was 0.807. The Cronbach’s alpha for four domains i.e. physical, psychological, social and environment were 0.765, 0.700, 0.812 and 0.780 respectively.

**Table 2:** Distribution of WHOQOL-BREF items’ response (n = 295).

WHOQOL-BREF items	Very Poor (1)	Poor (2)	Average (3)	Good (4)	Very Good (5)	Mean ± SD
**Overall QoL & Health**						
QoL Rating	3	14	86	114	78	3.84±0.90
Health Satisfaction Status	6	20	90	124	55	3.68±0.92
**Physical Domain**						
Q3 Physical Pain	12	39	101	85	58	3.46±1.07
Q4 Medication Need	10	31	70	87	97	3.77±1.11
Q10 Everyday Energy	4	32	83	95	81	3.73±1.02
Q15 Get around Ability	1	14	33	90	157	4.31±0.87
Q16 Sleep Satisfaction	11	29	80	121	54	3.60±1.01
Q17 Daily Activities	4	29	116	112	34	3.48±0.87
Q18 Work Capacity	8	11	119	118	39	3.57±0.86
**Psychological Domain**						
Q5 Enjoying Life	1	42	113	96	43	3.46±0.92
Q6 Meaningful Life	5	23	72	104	91	3.85±1.00
Q7 Ability to Concentrate	3	37	115	111	29	3.42±0.86
Q11 Body Appearance	9	46	81	73	86	3.61±1.14
Q19 Self Satisfaction	9	13	80	124	69	3.78±0.95
Q26 Negative Feelings	32	77	140	30	16	2.73±0.97
**Social Domain**						
Q20 Relationship Satisfaction	16	21	75	94	89	3.74±1.12
Q21 Sexual Satisfaction	36	24	85	62	88	3.48±1.32
Q22 Social Support	29	29	82	76	79	3.49±1.25
**Environmental Domain**						
Q8 Life Safety	12	18	77	111	77	3.75±1.03
Q9 Healthy Environment	21	40	108	96	30	3.25±1.04
Q12 Financial Satisfaction	14	40	105	90	46	3.38±1.05
Q13 Informational Sources	6	25	89	129	46	3.62±0.91
Q14 Leisure Activities	36	100	79	57	23	2.76±1.13
Q23 Living Place	23	21	59	90	102	3.76±1.21
Q24 Healthcare Satisfaction	18	43	72	84	78	3.54±1.19
Q25 Transport Satisfaction	29	44	52	56	114	3.61±1.37

Table 3 presents the mean HRQoL scores for all the four domains of WHOQOL-BREF among the study respondents. The mean score for the physical health domain was 62.44±15.36. Mean scores for the psychological domain, social relationships domain, and environment domain were 67.84±15.54, 64.27±26.28 and 63.45±17.66, respectively.

**Table 3:** Mean HRQoL scores for four domains of WHOQOL-BREF.

Domains	HRQoL Scores (Mean±SD)
Physical Domain	62.44±15.36
Psychological Domain	67.84±15.54
Social Relationship Domain	64.27±26.28
Environmental Domain	63.45±17.66

Table 4 shows the correlations between four different domains of WHOQOL-BREF. Based on the observed findings, statistically significant positive correlations were noted between all four domains of WHOQOL-BREF. There was also a statistically significant positive correlation between the first two questions of WHOQOL-BREF i.e. QoL and overall health satisfaction status and scores obtained from different domains. The strength of correlation among QoL and overall health status was moderately-strong (Spearman’s r >0.4), whereas four domains showed low, moderate, strongly-moderate and strong correlations (Spearman’s r ranged 0.155 to 0.627).

**Table 4:** Correlation coefficients in overall health and domains of WHOQOL-BREF.

		QoL	Overall Health	Physical Domain	Psychological Domain	Social Domain	Environmental Domain
**QoL**	Correlation (r)	1					
	Sig. (2-tailed)						
**Overall Health**	Correlation (r)	0.424	1				
	Sig. (2-tailed)	<0.001					
**Physical Domain**	Correlation (r)	0.472	0.416	1			
	Sig. (2-tailed)	<0.001	<0.001				
**Psychological Domain**	Correlation (r)	0.348	0.391	0.530	1		
	Sig. (2-tailed)	<0.001	<0.001	<0.001			
**Social Domain**	Correlation (r)	0.242	0.155	0.513	0.301	1	
	Sig. (2-tailed)	<0.001	<0.001	<0.001	<0.001		
**Environmental Domain**	Correlation (r)	0.487	0.376	0.627	0.494	0.481	1
	Sig. (2-tailed)	<0.001	<0.001	<0.001	<0.001	<0.001	

Table 5 represents correlation coefficients and the bivariate relationship between demographic variables and the domain scores. Statistically, a significant difference was observed between the scores of various age groups, marital status, education level, comorbidities other than CVDs against the psychological domain (*p*≤0.05). Patients in the age group of ≥30 years had significantly higher HRQoL scores in their psychological domain (69.48±14.74) than those in <30 years (65.38±16.43). A statistically significant difference was observed between the scores of marital status and the psychological domain (*p* = 0.045). A significant difference was also observed in education levels and psychological (*p* = 0.002) and environment domains (*p* = 0.016). Patients having primary and secondary education had significantly lower HRQoL in both psychological (54.00±13.75) and environment domain (50.20±17.45) than higher educated patients, respectively. Employed or business-doing patients had significantly higher HRQoL scores in the environmental domain (63.95±17.94) than non-workings (62.64±17.25). Patients with comorbidities other than CVDs were having compromised HRQoL in all four domains and QoL and overall health satisfaction status than those without any comorbidity (*p*-value ranged from 0.000 to 0.165).

**Table 5:** Comparison of WHOQOL-BREF mean scores, standard deviations, and significance levels based on sociodemographics.

Variable				Domains		
	QoL	Health Satisfaction	Physical	Psychological	Social	Environmental
**Gender**						
Male	3.71±0.97	3.62±0.95	62.34±15.33	68.13±15.36	64.68±24.95	63.18±17.86
Female	3.92±0.84	3.72±0.90	62.50±15.42	67.66±15.69	64.02±27.12	63.61±17.59
**P Value**	**0.045**	0.362	0.932	0.801	0.834	0.838
**Age**						
< 30 Years	3.93±0.90	3.72±0.87	64.19±15.34	65.38±16.43	62.60±25.40	63.38±18.29
≥ 30 Years	3.79±0.90	3.65±0.95	61.27±15.31	69.48±14.74	65.38±26.87	63.49±17.28
**P Value**	0.189	0.503	0.110	**0.026**	0.374	0.961
**Marital Status**						
Single/Separated	3.77±0.96	3.60±1.03	60.46±15.51	70.10±14.95	65.10±27.91	64.62±18.21
Married	3.89±0.86	3.73±0.84	63.70±15.18	66.39±15.78	63.73±25.26	62.70±17.31
**P Value**	0.265	0.207	0.078	**0.045**	0.664	0.362
**Education Level**						
Primary and secondary	3.20±1.03	3.50±0.52	54.00±13.75	52.60±7.87	63.10±16.55	50.20±17.45
Higher secondary and above	3.87±0.89	3.69±0.93	62.73±15.35	68.37±15.48	64.31±26.58	63.91±17.52
**P Value**	**0.021**	0.520	0.077	**0.002**	0.886	**0.016**
**Work Status**						
Not working	3.84±0.90	3.71±0.90	63.56±15.68	69.57±15.48	65.77±25.02	62.64±17.25
Job/Business	3.85±0.90	3.66±0.93	61.73±15.16	66.74±15.52	63.32±27.07	63.95±17.94
**P Value**	0.537	0.437	0.128	0.321	0.610	0.936
**Comorbidities other than CVDs**						
Yes	3.74±1.04	3.48±0.99	58.18±16.00	60.73±17.12	57.98±27.15	58.79±18.15
No	3.90±0.81	3.79±0.86	64.72±14.55	71.65±13.17	67.64±25.24	65.94±16.92
**P Value**	0.165	**0.006**	**<0.001**	**<0.001**	**0.002**	**0.001**
**Warfarin Indication**						
AF/Valve replacements	3.81±0.90	3.69±0.91	61.54±16.09	66.76±15.24	64.98±26.26	62.76±17.91
DVTs/PEs	3.87±0.90	3.67±0.93	63.17±14.75	68.72±15.78	63.68±26.37	64.01±17.49
**P Value**	0.543	0.906	0.363	0.283	0.673	0.548
**Warfarin Duration**						
< 1 Year	3.83±0.93	3.70±0.96	60.87±17.11	67.04±16.50	60.95±28.53	62.85±19.14
≥ 1 Year	3.85±0.88	3.66±0.89	63.60±13.85	68.43±14.81	66.74±24.27	63.89±16.51
**P Value**	0.817	0.729	0.131	0.450	0.061	0.619

Table 6 shows correlations between demographic variables vs different domains of WHOQOL-BREF. Based on the observations, statistically significant positive and negative correlations were noted between various demographic variables and four domains of WHOQOL-BREF. Statistically significant (positive and negative) correlation between the first two questions of WHOQOL-BREF i.e. QoL and overall health satisfaction status and demographic variables were noted. The strength of correlation among demographic variables and various domains of WHOQOL-BREF showed low, moderate and strongly-moderate positive and negative correlations (Spearman’s ‘r’ ranged from -0.117 to 0.335).

**Table 6:** Correlations between demographic variables and different domains of WHOQOL-BREF.

Variables	Correlations	QoL	Overall Health	Physical Domain	Psychological Domain	Social Domain	Environmental Domain
**Gender**	Correlation (r)	0.111	0.053	-0.011	-0.006	0.001	0.009
	*p* value	0.056	0.368	0.853	0.913	0.984	0.883
**Age (in Years)**	Correlation (r)	-0.079	-0.036	-0.102	0.122	0.070	-0.006
	*p* value	0.176	0.535	0.082	**0.036**	0.232	0.915
**Marital Status**	Correlation (r)	0.056	0.054	0.099	-0.118	-0.046	-0.055
	*p* value	0.339	0.360	0.091	**0.042**	0.431	0.346
**Education**	Correlation (r)	0.123	0.054	0.117	0.208	0.036	0.144
	*p* value	**0.035**	0.369	0.044	**<0.001**	0.535	**0.014**
**Work Status**	Correlation (r)	0.000	0.029	0.057	0.092	0.040	-0.033
	*p* value	0.999	0.621	0.325	0.116	0.498	0.577
**Diseases other than cardiac**	Correlation (r)	0.052	0.146	0.196	0.308	0.166	0.185
	*p* value	0.369	**0.012**	**0.001**	**<0.001**	**0.004**	**0.001**
**Warfarin Indication**	Correlation (r)	0.045	-0.002	0.061	0.073	-0.028	0.031
	*p* value	0.446	0.972	0.297	0.212	0.632	0.596
**Duration of therapy**	Correlation (r)	0.016	-0.044	0.053	0.030	0.100	0.011
	*p* value	0.787	0.542	0.368	0.608	0.087	0.845

Table 7 represents the linear regression analysis which revealed that for any change in marital status from single/separated to married, a score of 0.109 is increased for the physical domain (p<0.05) when adjusted for other demographic variables. Similarly, a change in education level from primary to secondary/higher was associated with an increase of 0.089 in the score for the psychological domain (p<0.05) keeping other demographic variables as constant. Furthermore, a change in comorbidity status, i.e., having no cardiovascular disease as comorbidity as opposed to having it, it would increase the scores by 0.153, 0.267, 0.143 and 0.127 in the physical, psychological, social and environmental domains (p<0.05) when adjusted for other patient variables. Furthermore, for a unit increase in the QoL score, it would increase to 0.496, 0.358, 0.280 and 0.487 in scores in the physical, psychological, social and environmental domains (p<0.05) when adjusted for other demographic variables. The variables of age and duration of warfarin therapy were not significant (p>0.05).

**Table 7:** Linear regression model.

Variables	Domains											
	Physical			Psychological			Social			Environmental		
	Coefficient (β)	p-value	VIF	Coefficient (β)	p-value	VIF	Coefficient (β)	p-value	VIF	Coefficient (β)	p-value	VIF
**Age (**< 30 Years vs ≥ 30 Years)	NA	---	---	0.060	0.336	1.50	NA	---	---	NA	---	---
**Marital status** (Single/Separated vs Married)	0.109	0.032	1.04	-0.052	0.408	1.48	NA	---	---	NA	---	---
**Education Level** (Primary and secondary vs Higher secondary or above)	0.031	0.534	1.05	0.089	0.032	1.05	NA	---	---	0.059	0.252	1.04
**Comorbidities other than CVDs** (Yes, vs No)	0.153	0.003	1.04	0.267	<0.001	1.05	0.143	0.011	1.01	0.127	0.013	1.02
**Warfarin Duration** (< 1 Year vs > 1 Year)	NA	---	---	NA	---	---	0.103	0.065	1.00	NA	---	---
**QoL**	0.496	<0.001	1.03	0.358	<0.001	1.03	0.280	<0.001	1.00	0.487	<0.001	1.01

Model fitness for different domain: ***Physical*** = *ANOVA (F = 30*.*54*, *p =* <0.001*); R*^*2*^ *= 0*.*296 and adjusted R*^*2*^ *= 0*.*286);* ***Psychological*** *= ANOVA (F = 18*.*96*, *p =* <0.001*); R*^*2*^ *= 0*.*248 and adjusted R*^*2*^ *= 0*.*235);* ***Social*** *= ANOVA (F = 12*.*26*, *p =* <0.001*); R*^*2*^ *= 0*.*113 and adjusted R*^*2*^ *= 0*.*103) and* ***Environmental*** *= ANOVA (F = 36*.*11*, *p =* <0.001*); R*^*2*^ *= 0*.*272 and adjusted R*^*2*^ *= 0*.*264)*

## Discussion

In the past decade, HRQoL is an emerging concept and an important treatment outcome parameter to assess patients’ general health state, treatment efficacy, and overall disease management [37]. This study determines the HRQoL of warfarin patients in four different domains of WHOQOL-BREF, together with overall health satisfaction and the factors responsible for such HRQoL outcomes. The severity of the chronic diseases demands healthcare practitioners to pay due attention to the HRQoL of the affected individuals. To the best of our knowledge, to date, this is the first-ever study done in Pakistan regarding HRQoL of warfarin patients using WHOQOL-BREF thus there are no studies evident as a cross‐reference to this study.

Researchers from different parts of the world explored different aspects of HRQoL among patients on anticoagulation therapy [38–43]. Limited access to medication use, annoyance, the burden of the CVDs, and both positive and negative psychological impacts are the significant concerns affecting HRQoL among patients on anticoagulation therapy [38–40]. The occurrence of a bleeding episode in patients on warfarin may cause a substantial decrease in their general health and overall health status [44–46]. In a study done by Abubaker et al., the HRQoL of patients on anticoagulant therapy was greatly associated with socio-demographic and clinical variables. They studied patients’ knowledge, satisfaction, and adherence to oral anticoagulant therapy using Oral Anticoagulant Knowledge (OAK) test. They determined that the intensity of bleeding events, the presence of comorbidities, various drug interactions, education level differences, patient’s age factors, and total therapy duration have a direct influence on the HRQoL [38].

The study population was categorized into two main age groups i.e. <30 years and ≥30 years of age because in the pilot study most of the warfarin patients visiting the study sites were ≥30 years of age with fewer exceptions, that’s why the cut-off was set at 30 years of age. This study indicated that younger patients had considerably better HRQoL than elders in the physical domain, whereas in psychological, social, and environmental domains, elderly patients had better HRQoL than youngers. The results of our study in terms of elder patients having better HRQoL were similar to the earlier two studies that discussed HRQoL among older patients but in different diseases [25, 47]. Our study findings could be endorsed to the fact that older people may have a better feeling and understandings of the meanings of social, psychological, and environmental life, so maybe they are more satisfied with their lives despite that they are on warfarin [41]. Furthermore, elders may embrace their diseases as a challenge and started living a more satisfactory life than youngers. Similarly, the youngers may also find their diseases as part of their lives and consider them a less challenging and life-long phenomenon, so they started living more satisfactory and confidently in terms of the physical domain [25, 47]. However, another previous study on warfarin patients did not describe any significant association between HRQoL scores and their age [48]. Our findings are contrary to Casais et al, where they found out that younger patients had a better satisfaction with their anticoagulant therapy as compared to the older patients [25]. These differences could be due to different research tools used, varied patients’ characteristics, lifestyle changes, and bleeding disorders. Gadisseur et al. reported that different factors like modalities of treatment plans, self-handling of the medicines, and counseling by specialized anticoagulation clinic staff are important influencing parameters affecting patients HRQoL while they are on warfarin [48].

In the current study, among the four domains of WHOQOL-BREF, the highest mean score (satisfaction level) was noted for the psychological domain (67.84±15.54) than the rest of the domains, which may be due to adequate healthcare facilities, body appearance, no negative feelings, more positive feelings, a greater level of self-esteem, high religious activities, spiritual applicability and personal beliefs [41]. Moreover, the lowest mean score (satisfaction level) was observed for the physical domain (62.44±15.36) among all domains, indicating compromised activities of daily living, more dependence on medicinal substances and medical aids, less mobility, and more fatigue, discomfort, and less work capacity. On the other hand, acceptable mean scores were observed for social and environment domains (64.27±26.28 and 63.45±17.66 respectively) showing good personal relationships, greater social support, satisfactory sexual activities, freedom in religious activities, and frequent access to cheap and convenient transportation [41–43].

On the contrary to the findings of Saad et al., our study determined a significant association of various sociodemographic factors with HRQoL among patients on warfarin [44]. We observed a significant effect of gender on HRQoL among patients on warfarin. It was interesting to note that females seemed more satisfied in overall health satisfaction states, physical domain, and environmental domain as compared with the males. Similarly, males appeared to be more satisfied in psychological and social domains as compared with females. These results are contrary to another study where investigators observed no difference in HRQoL scores between males and females [45]. These differences are may be due to different populations, different study sites, and different research tools used, i.e., they used SF-36 and Perceived Stress Scale, whereas we used WHOQOL-BREF. Our study results regarding gender differences are in accordance with Sayin et al. that the males had overall less HRQoL than the females [41, 46].

In the past, mixed findings were observed as in some other studies, married patients had higher HRQoL, whereas few studies reported that marital status did not have any effect on HRQoL [10, 37, 49, 50]. Our study also confirmed that married patients had higher HRQoL in QoL, overall health satisfaction states, and the physical domain, whereas unmarried or singles had better HRQoL in other domains. This could be because Pakistan has an extensive family structure where unmarried patients get adequate financial support and emotional backups from their parents and families. This is in contrast to various other countries and cultures, where support is often linked to marriages.

In literature, the association between education levels and HRQoL among chronically ill patients is well studied [37, 41]. According to our study, statistically significant differences were observed between the well and the poorly educated patients. Higher education is often linked with self-interest, better dosage understandings, high treatment compliance and medication adherence [51]. The findings of our study confirmed that the education level had a significant effect on overall HRQoL among patients on warfarin in Pakistan. In all four domains of HRQoL and overall health satisfactory states, much better scores were observed among highly educated patients than those having a primary level of education [52]. In general, highly educated patients were reported to live longer and enjoy better health status as compared to the less educated patients [41, 52, 53]. Our study results are similar to Casais et al, where study participants with higher education levels had better HRQoL [25]. In contrast, our study results are opposite to two other studies where no significant differences were found among education levels and HRQoL [37, 40].

In this study findings, employed/self-employed individuals scored comparatively better scores in three domains (physical, psychological, and social) of the WHOQOL-BREF than those who were not working, but the results were not statistically significant (p>0.05). These findings are contrary to the findings of Joshi et. al, in terms of environment domain and overall general health satisfaction states [37]. The differences in physical, social and psychological domains are may be due to differences in age groups, gender, educational status, ethnicity, marital status, employment, the environment of the study population, family income and duration of illnesses. Our study findings maybe because of the better access to the financial resources (employment), type of healthcare they received, access to the opportunities for acquiring up-to-date information regarding warfarin. These results were also consistent with that of Yang et al. and Casais et al., who presented significantly better scores for their respondents in the environment domain [25, 54]. Employment or having self-business can significantly improve HRQoL scores in physical, psychological, social, and environmental domains as in the studies conducted by Casais et al. [25] and Sathvik et al. [55]. Our findings in the social domain are contrary to Yang et al findings where they found low scores in the social domain particularly dissatisfaction with sexual life [54]. The reason for both opposite findings may include different disease states, cultural backgrounds, healthcare policies, access to quality transportation, religious matters, and satisfaction with healthcare facilities. Another contributing factor regarding the social domain may be that patients spend more time with their families and friends that may positively affect their personal and social relationships. Undeniably, better income appears to be a unique predictor of improved HRQoL [37]. According to the findings of another study, income was significantly associated with improved psychological and environmental domain scores [9, 55]. These findings are not surprising as high earning patients can easily adopt better treatment options to fulfill their healthcare needs as compared to those who are neither businessmen nor employed [25, 41].

In our study, better HRQoL was observed in patients with no comorbidity than those who were affected by various comorbidities other than CVDs. In all four domains of HRQoL and overall health satisfactory states, much better scores (*p*<0.05) were present in the patients without any comorbidity than those who were facing comorbidities. Our results are in agreement with a study done by Gadisseur et al., where they also found similar results [48]. In bivariate analysis, our results illustrate that the duration of warfarin usage had a significant effect on all domains of the WHOQOL-BREF. Patients had greater HRQoL who were on warfarin for more than a year than those who were on less than a year. These results are similar to Gadisseur et al., where they also found statistically significant differences among their respondents regarding the duration of warfarin utilization [48]. The linear regression model confirmed that marital status, education level, comorbidities other than CVDs, and general QoL variables are pure predictors (after adjusting confounders) against different domains the WHOQOL-BREF in determining HRQoL among warfarin patients in Pakistan.

In developing countries like Pakistan, warfarin yet is the drug of choice for various CVDs because of its therapeutic benefits and perceived cost-effectiveness [56, 57]. Conversely, warfarin’s optimum therapy management poses a greater challenge to the healthcare staff because of its varied dose-response, narrow therapeutic index, multi-factorial drug-food interactions and the need for adequate dose adjustments and monitoring [57, 58]. In developed countries, such challenges are dealt with by devising innovative treatment strategies like advanced anticoagulation clinics, patients’ self-monitoring, and various computer-aided adherence and treatment plans [56–58]. Generally, the total period and the treatment patterns of chronic illnesses like patients on longer warfarin requires longer treatment durations. In countries other than developed, the majority of them are facing a lot of challenges in providing optimum healthcare facilities to their population [1,2]. Though these days in Pakistan, some of the healthcare costs for chronic diseases are borne by the government, but still better treatment plans, adherence to the medications, compliance with the lifestyle modifications, awareness of the drug regimen are greater contributing factors affecting HRQoL of the patients on warfarin.

Though currently, Pakistan is facing some thwarting challenges in terms of economic, social, and political areas, yet lack of these resources does not mean people always die because of poverty or lack of healthcare facilities. Indeed, the quality healthcare in Pakistan has a high price tag, and good quality healthcare facilities are mostly available to the elite class in private healthcare set-ups, yet these days, much better facilities are being offered in public hospitals and clinics where patients are somehow more satisfied than the past. Both federal and provincial governments are trying their level best to overcome healthcare challenges to provide optimal healthcare facilities to the public. Definitely, lack of advanced healthcare facilities and less financial resources do have their impact on the patients’ overall HRQoL but from the results of our study, HRQoL among warfarin patients in Pakistan was not that bad as were the rumors. Moreover, in Pakistan, currently, various policies are also underway for the implementation of Minimum Service Delivery Standards (MSDS) for the provision of safe medicines, better patient care, improving cleanliness and hygiene conditions of the hospitals and clinics, infection control strategies and provision of the required healthcare staff, that will further improve the HRQoL of the patients especially in chronic diseases.

## Conclusion

The findings from this study confirm that the WHOQOL-BREF research tool is a reliable instrument to measure HRQoL among warfarin patients in Pakistan (Cronbach Alpha 0.898). From the obtained data, it is evident that warfarin patients in Pakistan enjoy overall satisfactory HRQoL in all domains of the WHOQOL-BREF, although some of the variables showed a relatively moderate or lower HRQoL in some domains. This lower HRQoL is may be due to non-acceptance of treatment pattern by the patients, inappropriate therapeutic plans, non-compliance, increased indirect medical costs, increased direct non-medical costs, inability to work, and increased overall living expenses. The findings of this study are an imperative contribution in literature for the understanding of the effect of warfarin on overall HRQoL among patients on warfarin in Pakistan. This study is also novel and first of its kind because few studies are done in various countries to measure HRQoL of warfarin patients, but none is done in Pakistan using the WHOQOL-BREF. Since most of the information evident in the literature regarding the relationship between HRQoL and anticoagulation control of the warfarin comes from other countries, whose extrapolation for the Pakistani society is limited by cultural, religious, socio-economic differences and the way the healthcare system is developed and managed in Pakistan.

### Limitations of the study

As the majority of the HRQoL questionnaires, the WHOQOL-BREF is also a self-reported study tool and in cases of illiterate patients, the tool is filled with the help of patients’ caregivers, nurses or the investigators themselves that may report some biases. In this scenario, data reporting biases may have acted as confounding factors in our study. One of the limitations noted for our study was that there was no control group to make applicable comparisons for the findings obtained. Another limitation of this study was its cross-sectional design as in cross-sectional studies, generalizability is often limited by the sampled population because sample size requirements are often very large. The cross-sectional studies, on the other side, also have the potential for selection bias where they cannot exactly determine the causal relationship between the studied variables and the outcomes obtained. This study also did not discuss the HRQoL of warfarin patients having uncontrolled INRs i.e. bleeding risks, which is another limitation of this study. As this study used purposive sampling technique, which also has some limitations like errors in judgment by the researchers, less reliability and high levels of bias, and research findings can not be too generalized. Despite some limitations, the findings of this study shed significant light on the overall status of HRQoL among warfarin patients in Pakistan. The findings of this study could help physicians, pharmacists, allied healthcare professionals, and the family members of the patients to better understand the physical, psychological, social and environmental problems patients usually face while on warfarin. This, in return, will definitely help and encourage them to provide more physical, psychological, and social support to their patients.

## Supporting information

S1 Raw data (SAV)
